# Potential Drug-drug Interactions among Hospital Discharge Prescriptions in a Tertiary Care Centre of Nepal: A Descriptive Cross-sectional Study

**DOI:** 10.31729/jnma.7065

**Published:** 2022-02-28

**Authors:** Bijay Bhandari, Pratik Lamichhane, Dipendra Yadav, Sangha Ratna Bajracharya

**Affiliations:** 1 Department of Clinical Pharmacology, Institute of Medicine, Tribhuvan University, Kathmandu, Nepal; 2 Maharajgunj Medical Campus, Institute of Medicine, Tribhuvan University, Kathmandu, Nepal

**Keywords:** *drug interactions*, *Nepal*, *patient discharge*, *software*

## Abstract

**Introduction:**

In our setup, potential drug-drug interactions are overlooked in routine clinical practice. In general, most of the discharges are handwritten in the developing world, and the discharge prescriptions are not checked with the database for potential drug-drug interactions checker. This study aimed to determine the prevalence of potential drug-drug interactions in the prescribed drugs in clinical practice in a tertiary care centre of Nepal.

**Methods:**

A descriptive cross-sectional study was conducted in a tertiary care center from October 2019 to December 2019. Ethical approval was taken from the Institutional Review Committee (Reference number: 394(6-11)E2/075/76). Through simple random sampling, the data about drug prescription was collected from the patient discharge records of the Department of Internal Medicine. The potential drug interactions were checked by using Lexicomp® drug interactions. Data was analysed using Statistical Package for the Social Sciences version 20.0. Point estimate at 95% Confidence Interval was calculated along with frequency, percentage, mean, standard deviation and mode.

**Results:**

Among 382 discharge prescriptions, the prevalence of potential drug-drug interactions was 299 (78.3%) (74.1-82.4 at 95% Confidence Interval). A total of 1519 drug interactions with a mean of 5.08±3.89 drug interactions per prescription was identified. The major, moderate and minor drug-drug interactions according to the severity were found to be 163 (10.7%), 1162 (76.5%), and 178 (11.7%) respectively.

**Conclusions:**

The prevalence of potential drug-drug interactions is high among the patients on discharge compared to similar studies. Use of drug-drug interactions checker databases before discharge with computer-based discharge prescriptions is recommended.

## INTRODUCTION

Drug-Drug Interaction (DDI) refers to the modification of response to one drug by another when they are administered simultaneously or in quick succession.^1^ The alteration is mostly quantitative, i.e., the response to a drug is either increased or decreased in intensity. Drug interactions represent 3-5% of in-hospital Adverse Drug Reactions (ADRs). DDIs can result in hospital admission, varying from ineffective treatment to life threatening adverse drug events. The proportion of hospital admissions due to DDIs ranges from 0% to 3.8%.^2,3^ Polypharmacy, higher age of the patient, and presence of co-morbidities considerably contribute to one or more drug interactions.^4^

After discharge from the hospital, patients are not under direct supervision for their pharmacotherapy, so anticipation of potential DDIs (pDDIs) plays a major role in patient care.

This study aims to determine the prevalence of potential drug-drug interactions in discharge prescriptions from the internal medicine ward of a university teaching hospital.

## METHODS

A descriptive cross-sectional study was conducted on in-patients discharge prescriptions from different units of the department of internal medicine of Tribhuvan University Teaching Hospital (TUTH), Nepal. The data were collected retrospectively by reviewing the medical chart of the hospital over a period of three months from October 2019 to December 2019. Ethical approval was taken from the Institutional Review Committee of the Institute of Medicine (Reference number: 394(6-11)E^2^/075/76).

The sample size was calculated using the formula:

n = Z^2^ × p × q / e^2^

  = (1.96)^2^ × 0.5 × (1-0.5) / 0.05^2^

  = 385

Where,

n = minimum required sample size
Z = 1.96 at 95% Confidence Interval (CI)
p = prevalence of pDDI taken as 50% for maximum sample size
e = margin of error, 5%

A total of 1203 patients discharged with prescriptions as per our inclusion criteria was taken as a sampling frame and their inpatient numbers were collected from the hospital register. The in-patient numbers were randomly selected using Microsoft Excel 2016 to generate a sample population using simple random sampling technique. Medical records of the sample population were traced for discharge prescriptions. Informed consent was not applicable due to the retrospective nature of the data collection.

Data was collected by investigators in the data abstraction form and entered into an electronic format after being verified by the co-investigator. The data on patient's age, sex, diagnosis, co-morbidities, prescribed medicines, and length of stay were collected. Handwritten prescriptions where spellings were illegible were clarified by the prescribers of the concerned unit. Discharge records of patients with two or more drugs who stayed for one or more days as inpatient in the internal medicine ward of TUTH during the study duration were included. Discharge prescriptions of the patients admitted in the medicine ward due to unavailability of beds in other specialties, prescriptions with topical drugs, probiotics and nutritional supplements only, patients who left against medical advice (LAMA), mortality cases, patients who were referred or transferred elsewhere without a discharge prescription were excluded. Potential DDIs in the prescriptions were identified by using Lexicomp™ drug interaction software (available online in the Uptodate website and Application).

The Lexicomp™ software database classifies drug interactions into minor, moderate and major with an increasing degree of severity of interactions. The minor severity of interactions produces effects that are tolerable and do not require medical intervention. The moderate severity of interactions causes considerable adverse effects and requires medical intervention. The major severity of interactions produces effects that may result in hospitalisation, death, permanent injury, or therapeutic failure. Similarly, the risk rating of the drug interactions was sorted into five categories: A, B, C, D and X. Drug interactions which fall under the categories A, B and C do not require therapy modification but may require constant monitoring. However, interactions belonging to group D shall be addressed by considering therapy modification. Likewise, if potential DDI falls under risk rating X, such a drug combination is contraindicated and should be avoided at any cost.

Data analysis was performed using Statistical Package for the Social Sciences (IBM SPSS Statistics for Windows, Version 20.0. Armonk, NY: IBM Corp.). Point estimate at 95% Confidence Interval was calculated along with frequency, percentage, mean, standard deviation and mode.

## RESULTS

A total of 401 discharge prescriptions collected, 19 were invalidated as per exclusion criteria. Among 382 patients' discharge prescriptions studied, 299 (78.3%) is the prevalence of pDDI during study period (74.1-82.4 at 95% Confidence Interval). The total number of drug interactions were 1519 with a mean of 5.08±3.89 pDDI per patient. Out of 382 prescriptions, 216 (56.5%) were prescribed to males and 166 (43.5%) were prescribed to females. The mean age of study population was 53.8±20.0 years. The majority of the sample consisted of patients admitted to the Department of Cardiology; 94 (24.6%) followed by the Department of Neurology; 86 (22.5%). The mean number of comorbidities was 2.6±1.4 per patient. A total of 2382 drugs were used in the sample population with a mean number of medications received being 6.2±2.7 per prescription (Table 1). The median duration of hospital stay was six days.

**Table 1:** Characteristics of the patients (n = 382).

Characteristics	n (%)
**Age (years)**	
<15	3 (0.8)
15-30	63 (16.5)
30-45	68 (17.8)
45-60	92 (24.1)
60-75	105 (27.5)
>75	51 (13.4)
**Sex**	
Male	216 (56.5)
Female	166 (43.5)
**Medical Units**	
Cardiology	94 (24.6)
Gastroenterology	51 (13.4)
Nephrology	67 (17.5)
Neurology	86 (22.5)
Pulmonology	84 (22.0)
**Comorbidities**	
0-2	213 (55.8)
3-4	124 (32.5)
5-6	45 (11.8)
**Medications**	
1-4	118 (30.9)
5-8	181 (47.4)
9-12	83 (21.7)

Six (1.6%) discharge prescriptions consisted of a single drug removing the possibility of drug-drug interactions. In addition to this, five patients were prescribed topical agents at discharge along with other medications. Among patients with at least one interaction, 100 (26.2%) patients had more than five potential DDIs. The top ten common interactions in the study population are illustrated below (Table 2).

**Table 2:** Top ten common pDDIs (n= 1519).

Drug Interactions	n (%)	Severity	Risk Rating
Aspirin + Clopidogrel	70 (4.6)	Moderate	C
Vitamin D3 + Calcium carbonate	34 (2.2)	Moderate	C
Clopidogrel + Pantoprazole	33 (2.2)	Major	C
Clopidogrel + Atorvastatin	29 (1.9)	Moderate	B
Cefpodoxime + Pantoprazole	27 (1.8)	Moderate	C
Furosemide + Aspirin	26 (1.7)	Moderate	C
Levothyroxine + Pantoprazole	22 (1.4)	Minor	B
Furosemide + Fluticasone/Salmeterol	20 (1.3)	Moderate	C
Iron + Pantoprazole	20 (1.3)	Minor	B
Prednisolone + Calcium carbonate	20 (1.3)	Moderate	D

Among 299 cases with drug interactions, 170 (56.9%) were males and 129 (43.1%) were females. The distribution of pDDIs on the basis of age-groups, sex and different units of the department of Internal Medicine is illustrated below (Table 3).

**Table 3:** Age-wise, sex-wise and unit-wise distribution of drug interactions (n= 299).

Patient character	Number of patients with interactions n (%)	Number of pDDI n (%)
**Age (years)**		
<15	2 (0.7)	4 (0.3)
15-30	45 (15.1)	184 (12.1)
30-45	44 (14.7)	196 (12.9)
45-60	80 (26.8)	377 (24.8)
60-75	85 (28.4)	524 (34.5)
>75	43 (14.4)	234 (15.4)
**Sex**		
Male	170 (56.9)	896 (59.0)
Female	129 (43.1)	623 (41.0)
**Medical Units**		
Cardiology	79 (26.4)	557 (36.7)
Gastroenterology	32 (10.7)	107 (7.0)
Nephrology	63 (21.1)	344 (22.7)
Neurology	55 (18.4)	221 (14.6)
Pulmonology	70 (23.4)	290 (19.1)
**Medications**		
1-4	55 (18.4)	90 (5.9)
5-8	161 (53.9)	644 (42.4)
9-12	83 (27.8)	785 (51.7)
**Comorbidities**		
0-2	148 (49.5)	571 (37.6)
3-4	108 (36.1)	598 (39.4)
5-6	43 (14.4)	350 (23.0)

**Figure 1:**
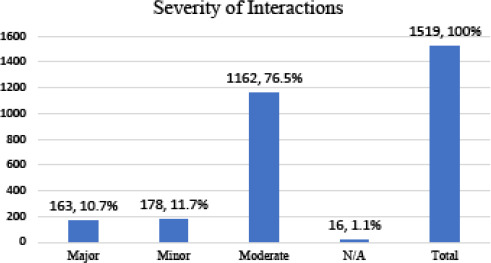
Severity of potential drug-drug interactions.

The major, moderate and minor pDDIs according to the severity were found to be 163 (10.7%), 1162 (76.5%), 178 (11.7%) respectively (Figure 1).

**Figure 2:**
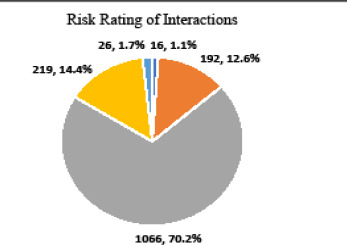
Risk ratings of potential drug-drug interactions (n= 1519).

pDDIs were also categorised according to the risk rating into A, B, C, D and X. The majority 1066 (70.2%) of the interactions fell under C category (Figure 2). Only 26 interactions (1.7%) fell under the X category.

## DISCUSSION

According to our study, the prevalence of potential DDIs among discharged prescriptions from the wards of Internal Medicine of TUTH is 78.3%. Compared with other studies done in patients at discharge, the prevalence of pDDIs in our study is higher. A prevalence of 52.91% has been reported among elderly patients discharged from tertiary centre of India.^5^ Likewise, a lower prevalence of 63% and 60% at discharge has been reported by the studies from Slovenia and Switzerland respectively.^6,7^ However, a similar prevalence of drug interaction; 78.8% has been found among ambulatory patients visiting outpatient department (OPD) in Mexico.^8^ In contrast, a study from Nepal on OPD patients showed a prevalence of 52.2% drug-drug interactions.^9^ A study among geriatric patients visiting OPD in India showed a prevalence of 83.25% which was attributed to a large number of drugs they received for multiple co-morbidities.^1^ Furthermore, another study from India on in-patients of a teaching hospital determined a prevalence of as high as 91%.^10^ This inconsistency in pDDI prevalence among the studies may be due to variation in study site, study population, pDDI checking software and drug-prescribing pattern.

Among patients with interactions, more than one thirds were above 60 years of age. Almost half of the total pDDIs were observed in patients above 60 years of age. Increasing age of the patient has been found associated with increasing number of potential drug interactions by various studies^1,6-8^ Males account for 59% of total interactions which is supported by a higher prevalence of pDDIs observed among males in the literature.^1,9^

Our study revealed the mean number of medications per prescription as 6.2±2.7. Former studies have also shown similar findings, with a study done at India showing the mean number medications as 6.5±2.2 and the study done in Ethiopia showing the mean number medication prescribed to elderly patients as 6±4 per patient.^1,11^ In patients with 5 drugs, pDDIs were 6.7% while in case of seven drugs, pDDIs were found to be more than double (14.4%). A large proportion (94.1%) of the interactions were observed in the patients who were prescribed five or more medications. Use of multiple medicines in the patients has been recognized as one of the factors contributing to pDDIs by the literature.^1,4,6,8,12^

In terms of severity-levels of pDDIs, in this study, moderate-pDDIs were mostly observed followed by minor-pDDIs. A study among OPD patients of Nepal has also described a high prevalence of moderate severity of potential drug-drug interactions. This finding is consistent with a number of studies from different places around the world.^5-7,10,12^ The major pDDIs constituted 10.7% of all interactions identified in the study. Similar to our finding, 12.2% of interactions at discharge were of major severity at a university hospital of Switzerland.^7^ However, a study from South India reported 2% prevalence of major pDDIs at discharge.^5^

The risk rating of the most interactions in the study were classified as category C. The interactions belonging to category C are the most common pDDIs according to other studies too. More than two-thirds of interactions in Mexican as well as Indian patients were classified into the same category.^1,8^ It is gratifying to know the category X drug interactions accounts for 1.5% of all interactions which is lesser than 3.0% and 3.8% as reported by Shetty V, et al. and Dubova SV, et al. respectively.^1,8^

Assessment for pDDIs on hospital discharge is crucial because about one-fifth of patients had potential major interactions on discharge, and half of these were due to the drugs introduced during hospitalization of the patients.^6^ After discharge from hospital, patients are not under direct supervision for their pharmacotherapy, so anticipation of pDDIs plays major role in patient care.

There are a few limitations to our study. Our study has not described the mechanism behind the potential drug-drug interactions. Moreover, risk factors for drug interactions among the patients were not analysed in the study. The study cannot precisely determine how many of the potential DDIs actually manifest clinically, leading to adverse events in the patients.

## CONCLUSIONS

The prevalence of potential drug-drug interactions observed among patients on discharge was similar to publisded literature. Use of drug-drug interactions checker databases before discharge with computer-based discharge prescriptions are recommended. The findings from this study will help to increase awareness of pDDIs so as to minimise the potential harm during an unsupervised phase of drug intake at home. This Conflict of Interest: None. baseline study can lead to further prospective studies to observe clinically evident drug-drug interactions outcome.
